# Enhancement of oral bioavailability of risedronate through xyloglucan rafts

**DOI:** 10.3389/jpps.2025.15525

**Published:** 2026-01-07

**Authors:** Nader I. Namazi, Rawan Bafail, Abdulkareem Ali Alanezi, Afaf F. Almuqati, Mohammed Salem Alshammari, Majed A. Alghamdi

**Affiliations:** 1 Department of Pharmaceutics and Pharmaceutical Industries, College of Pharmacy, Taibah University, Medina, Saudi Arabia; 2 Department of Pharmaceutics, College of Pharmacy, University of Hafr Al-Batin, Hafr Al-Batin, Saudi Arabia; 3 Department of Pharmaceutical Chemistry, College of Pharmacy, University of Hafr Al -Batin, Hafr Al-Batin, Saudi Arabia; 4 Department of Pharmacy Practice, College of Pharmacy, University of Hafr Al-Batin, Hafr Al-Batin, Saudi Arabia; 5 Department of Pharmaceutics, Faculty of Pharmacy, King Abdulaziz University, Jeddah, Saudi Arabia

**Keywords:** xyloglucan, risedronate, raft, bioavailability, *in vitro* dissolution

## Abstract

Bisphosphonates irritate the stomach and oesophagus and have a very limited absorption. The purpose of this study was to increase risedronate (RDN) oral bioavailability by causing a raft to form in the stomach. The creation of a raft prevents the irritation of the stomach and oesophagus caused by bisphosphonates. FTIR, TGA, and DSC were used to characterise the RDN, XLG, and the created formulation. In addition to a cell viability analysis utilising Caco-2 cells, the release of RDN was investigated in 0.1 N HCl, 0.5 N HCl, 1 N HCl, and simulated gastric fluid (SGF). For the pharmacokinetic investigation, the XR5 formulation and the Actonel® tablet were chosen as the test and reference formulations, respectively. Using a parallel design, twelve healthy albino rats were split into two groups, and blood samples were gathered for a whole day. RDN was distributed uniformly throughout the raft and demonstrated chemical stability by the FTIR. The formulation’s thermal stability was demonstrated by the TGA and DSC. At 20 min, the SGF showed a 99.97% RDN release. When compared to the RDN suspension, the pharmacokinetics revealed better RDN values from the XLG raft. The RDN from the recently developed XR5 has a better bioavailability than the Actonel® tablet.

## Introduction

Numerous conditions associated with Paget’s disease, cancer-induced hypercalcemia, postmenopausal osteoporosis, and bone resorption is treated with bisphosphonates. Drugs of BCS class III (high solubility and low permeability) have a limited permeability through the gastrointestinal mucosa, making it challenging to achieve the required bioavailability [1]. When taken orally as regular tablets, risedronate, a bisphosphonate that is a member of the BCS class III and is used in osteoporosis treatment, has an extremely low bioavailability. Unfortunately, there have been worries about oral bisphosphonate medication, notably risedronate, which has been connected to upper gastrointestinal injuries. According to reports of adverse experiences, oral bisphosphonate may cause severe intolerance to the upper gastrointestinal tract by irritating the local mucosa. In a small percentage of patients, this may result in undesirable esophageal experiences like oesophagitis, ulcers, and erosions [2].

Xyloglucan (XLG) is a polysaccharide derived from plants that is obtained from tamarind seeds. Its backbone chain is composed of (1–4)-β-D-glucan, with branches of (1–6)-α-D xylose that have been partially replaced by (1–2)-β-D-galactoxylose. XLG is composed of oligomers of monosaccharide, octasaccharide, and heptasaccharide with different numbers of galactose side chains [3]. XLG gels have the potential to be used for drug delivery via oral, intraperitoneal, ocular, and rectal routes. The gelation time of XLG is a few minutes. Its use in oral delivery takes advantage of the suggested slow gelation time (several minutes), which would allow *in situ* gelation in the stomach following the administration of a chilled solution of XLG orally. Ionic Cross-linking: In the presence of certain ions, many polysaccharides undergo a phase transition [4]. Polysaccharides that are ion-sensitive are the most commonly used. In the presence of different ions such as k+, Ca+, Mg+, and Na+, ion-sensitive polysaccharides such as carrageenan, gellan gum (Gelrite®), pectin, and sodium alginate undergo a phase transition. In the presence of various monovalent and divalent cations, various polysaccharides undergo gelation. Due to the interaction with the guluronic acid block in alginate chains, alginic acid undergoes gelation in the presence of divalent/polyvalent cations such as Ca2+. In the presence of a small quantity of K+, K-carrageenan forms hard, brittle gels, whereas i-carrageenan forms elastic gels primarily in the presence of calcium. An anionic polysaccharide, gellan gum, which is commercially available as Gelrite®, undergoes *in situ* gelling when mono- and divalent cations like calcium, magnesium, potassium, and sodium are present. Divalent cations, particularly calcium, have the potential to cause gelation of the xyloglucan [5].

The purpose of the study was to develop immediate release formulation because raft forming tablets immediately disperse and forms raft over the stomach contents. The bisphosphonates cause stomach irritation if the pH of the stomach is below 2.5. The raft forming tablets contain effervescent mixture that can neutralize the pH of the stomach above 3.5 for 30 min. Within these 30 min the drug can be release and absorbed from the upper portion of small intestine. The raft prevents the reflux of gastric contents to esophagus and prevents the irritation to esophagus [6]. The raft forming systems effectively utilized for the delivery of drugs through stomach [7]. The XLG raft is developed first time for the delivery of RDN according to our best of literature search. The XLG raft effectively raises the pH of the stomach above 3.5 and at that pH there is no irritation of the stomach lining. The drug is effectively absorbed from the upper part of small intestine due to the presence of penetration enhancer i.e., PEG 400. The developed rats were characterized using various physical and chemical parameters. The pharmacokinetics of the RDN was assessed using albino rats.

## Materials and methods

### Materials

Risedronate (RDN) was obtained as a gift sample from SAJA Pharmaceuticals, Jeddah, Saudi Arabia. Xyloglucan (XLG), HPMC E5, PEG 400, carboxymethyl cellulose (CMC), calcium carbonate, sodium bicarbonate, and citric acid were purchased from Sigma Aldrich Gmbh Darmstadt, Germany.

### Preparation of raft forming tablets

The raft forming RDN containing prompt delivery tablets were made using the wet granulation process. As indicated in Table 1, RDN, XLG, PEG 400, and other excipients were properly blended. RDN and other excipients were combined as a powder using a Sigma mixer. 2% (w/w) HPMC E5 in a 90% ethanol solution was used to granulate the powdered mixture. The granules were crushed with a Minipress MⅡ (Pharma Test, Hainburg, Germany)after being dried at 40 °C for 2 hours and passing through the 18-mesh screen [8].

**TABLE 1:** Composition of XLG raft forming formulations.

Code	RDN (%)	XLG (%)	CMC (%)	PEG 400 (%)	NaHCO_3_ (%)	Citric acid (%)	CaCO_3_ (%)
XR1	35.00	12.50	2.10	2.25	25.00	12.65	10.50
XR2	35.00	27.50	2.10	2.25	18.00	08.00	07.15
XR3	35.00	10.00	2.10	2.25	27.00	13.00	10.65
XR4	35.00	12.50	2.10	2.25	26.00	13.00	9.15
XR5	35.00	10.00	2.10	2.25	30.00	14.00	6.65
XR6	35.00	2.50	2.10	2.25	33.00	16.00	9.15
XR7	35.00	2.50	2.10	2.25	36.00	18.00	4.15
XR8	35.00	7.50	2.10	2.25	34.00	17.00	2.15
XR9	35.00	5.00	2.10	2.25	36.00	18.00	1.65

### Solubility studies

Solubility studies of the raft forming formulation contain XLG was evaluated using 0.1 N HCl and phosphate buffer having pH 5.8. The formulations (XR1 to XR9) was added to the solvent, vortexed and measure the concentration in the supernatant solution after the equilibrium reached [8].

### Disintegration time of tablets

To determine the disintegration time, a single tablet was placed in a 250 mL beaker with 120 mL of water at room temperature. The tablet was considered to have disintegrated if the gas surrounding it or its fragments stopped changing and was either dissolved or distributed in water so that there was no longer any agglomeration. Four more tablets underwent the same procedure [9].

### Hardness of tablets

This was done to account for the mechanical shock that tablets undergo during preparation, transportation, and storage. There is a lot of equipment available to measure tablet hardness. Tablet hardness was assessed using an Erweka hardness tester (Gmbh Germany) and expressed in Kg/cm^2^.

### Release of RDN from XLG rafts

The release of RDN from XLG raft-forming tablets was measured using a technique that was already used by Abbas and Hanif, described in 2017 [10]. The tablet was submerged in 50 mL of four distinct acidic media (SGF), 0.1, 0.5, and 1.0 N HCl solution in a 250 mL beaker [11]. At intervals of 5, 10, 15, 30, 45, and 60 min, 2 mL of sample was collected while the temperature was kept at 37 °C. In order to preserve the sink condition, the beaker containing the tablets for the dissolution research was filled with the same amount of new dissolution medium. In this study, a Shimadzu HPLC (20A series) system fitted out with a quaternary pump (LC-20AT), photodiode array (PDA) detector (SPD-M20A), auto-sampler (SIL-20ACHT), column oven (CTO-20AC), degasser (DGU-20A5R), and “Lab-Solution” software was employed. A Zorbax Eclipse plus C18 (4.6 × 250 mm, 5 μm particle size, Agilent, United States) was used as a column. Before being injected into the HPLC system for analysis, the samples were collected at regular intervals and filtered through a 0.22 m filter. At a flow rate of 1 mL/min, the analysis was performed using a mobile phase consisting of potassium phosphate buffer pH 2.5 and potassium edentate buffer pH 9.5 in a 50:50% v/v ratio. With 10% ortho-phosphoric acid, the pH of the mobile phase was adjusted to 6.8. The detection wavelength was adjusted at 263 nm. RDN had a retention time of 3.513 ± 0.3 min. A correlation coefficient (*R* ^*2*^) of 0.999 was obtained for the linearity parameter, which was investigated in the 20–200 µg/mL range. The recovery rate was found to be 99.92%. The calculated recovery percentage was 99.97%. The LOD and LOQ were 0.16 μg/mL and 0.11 μg/mL respectively. It was found that the precision value’s percentage relative standard deviation was less than 2%.

### Properties of xyloglucan raft

#### Acid neutralization capacity (ANC) within the raft

To determine how well the raft could retain the antacid and to provide the benefit of an antacid reservoir, the acid-neutralization capability of the raft was evaluated. The ANC value of the raft forming tablet was also evaluated. Two flasks with a 500 mL capacity were used. Reagent A was prepared by adding deionized water and 1 M HCl to flask 1, then heating it for 20 min at 37 °C in a water bath at 750 rpm. Reagent B was prepared by adding 0.5 M NaOH to flask 2 and heating it for 20 min at 37 °C at 600 rpm. The tablet was put into a beaker and was left there until the raft had fully developed. The raft was moved to a centrifuge tube following a wash with purified water (n = 3). After adding ethanol and a reasonable amount of filtered water at 4 °C, the mixture was centrifuged for 5 minutes at 5,000 rpm. The ethanol rapidly remove the water from the raft, this causes the raft solidify and raft was converted from fragile to more robust form which can be easily handled. After being removed from the centrifuge tube, the raft was heated in an oven at 40 °C. The raft powder was mixed with 150 mL of purified water in a conical flask. The flask was shaken with 30 mL of reagent A at 250 rpm for 60 s with the help of a shaker at 37 °C. Reagent B was introduced via a burette, and the titration analysis began. Changes in pH were checked by pH meter. The ANC was calculated using the equation below.

$$ANC=V-T\times 0.5\times \frac{total\;mass\;of\;raft\;\left(mg\right)}{weight\;of\;sample\;\left(mg\right)}$$

Where V is the volume of HCl (mL), and T is the volume of titer (mL).

#### Profile of neutralization

The neutralization profile of the raft is used to determine how well it can act as an antacid reservoir and provide protection against stomach acid; 150 mL of SGF was added to a beaker, and the temperature was maintained at 37 °C. Until the raft was completely formed, the tablet was placed in a beaker and left there. After the xyloglucan raft was transferred to a Büchner funnel, the media was disposed of. Three mL of 0.04 M HCl were added to the raft after 5 minutes of waiting. The resulting solution was thrown away. Following disposal, the raft was filtered using a 0.1 M HCl solution (n = 3), the collected solution was taken, and a pH was taken by a digital pH meter [12].

#### Swelling of xyloglucan raft

Then, in a beaker, 150 mL of SGF was added while keeping the temperature constant at 37 °C. Until the XLG raft was completely formed, the tablet was placed in a beaker and left there [13]. Using a spatula, the XLG raft was shifted to the Büchner funnel, where it was left until all of the supernatants had been removed. With the use of a spatula, the XLG raft was moved to the electronic weight scale and weighed and denoted by W_o_. The XLG raft was added to a plastic container containing 0.1 N HCl through a mesh and positioned on a shaker (orbit). After 10, 20, 30, 40, 50, and 60 min, the XLG raft was taken out of the plastic container, the extra water was drained, and it was weighed and given the W_1_ designation. The XLG raft was dried in an oven at 80 °C until its weight stayed constant, and the final weight was determined and designated W_2_. The process was repeated thrice (n = 3).

The following equation was used to estimate the swelling.

$$\%\;swelling\;of\;xyloglucan\;raft=\frac{W1-W2}{Wo}\times 100$$

### Floating lag time (FLT) total floating time (TFT)

FLT and TFT of the XLG raft were computed using the USP dissolution apparatus II (pharma test Hainburg, Germany) and 900 mL SGF pH 1.2 maintained at 37 ± 0.5 °C and 50 rpm. The time required for the raft to ascend to the surface and float was determined to be the FLT. The total time the XLG raft floats in the medium, including FLT, is called TFT. The process for FLT and TFT repeated thrice (n = 3).

### Permeation of RDN

The small intestine of albino rats was used in a penetration research. The upper portion of the small intestine was separated for the permeation research after the animal had been slaughtered. A newly dissected small intestine was mixed with the simulated intestinal fluid. A Franz diffusion cell with a vertical configuration was used for the permeation study. The Franz diffusion cell, which included a 9 mm orifice diameter, a flat ground joint, and a 5 mL receptor volume, was used. The thickness of the small intestine that was separated, cleaned, and placed in between the donor and receptor compartments was 0.54 mm. The diffusion cell’s donor compartment faced the surface of the small intestine. At regular intervals, 0.5 mL of samples were removed from the receptor compartment and filtered through a 0.45 μm membrane. To keep the sink state, 0.5 mL of the medium was added to the receptor compartment. The extracted sample was filtered, and 100 μL was then added to the HPLC apparatus to measure the amount that had permeated.

### Characterization of XLG rafts

By using Differential scanning-calorimeter DSC-60 (Shimadzu, Germany), FTIR spectrophotometer (Bruker Alpha, Germany), TGA analyzer, and diffractometer, Spectra of FTIR, DSC and TGA, XRD diffractograms of RDN, XLG and formulations were obtained respectively. The wavelength range obtained for the FTIR spectra was 4,000 to 1,000 cm^−1^. The samples for TGA and DSC were put in an aluminum pan and heated from 50 to 400 °C while being examined under a 100 mL/min nitrogen gas stream.

### Cell viability study (commercially purchased cells)

To evaluate the cellular toxicity of the XLG raft, an MTT assay on cell viability was conducted using the Caco-2 model. The commercially available Caco-2 cell line was purchased from The American Type Culture Collection, ATCC, located in Manassas, Virginia. In summary, 20% fetal bovine serum (FBS) and Caco-2 cells were cultivated in 96-well plates using Eagle’s Minimum Essential Medium (Catalog No. 30-2003). Following the assemblage of Caco-2 cells, medium, and FBS, the cells were cultured for 6 and 24 h in *Dulbecco’s Modified Eagle Medium* (*DMEM*) without FBS and containing 0.5% dispersions of various samples. Thereafter incubation, the samples were carefully and completely taken out and properly washed with phosphate-buffered saline thrice (Normal concentration of PBS; diluted to 1x). The cells were then incubated for a further hour with 500 µL of MTT solutions in FBS-free media (0.5 mg/mL) added to each well. Then the supernatants were removed, and 500 µL of dimethyl sulfoxide (DMSO) was used to solubilize the transformed dye. The resultant solution’s absorbance was measured at 570 nm. The following calculation was used to compute the percentage of cell viability;

$$\text{Cell viability }\left(\%\right)=\left(As÷Ad\right)\times 100$$

Then Following treatment with sample dispersions and DMEM, the absorbance is determined as As and Ad, respectively.

### Stability studies

Stability studies of XLG rafts were performed and ICH guidelines were followed strictly for 6 months. For the duration of the stability tests, the XR5 formulation was maintained at 40 °C and 75.5% relative humidity in a stability chamber. Samples of the RDN containing XLG rafts formulation were examined for disintegration time, tablet hardness, FLT, TFT, drug content, and release profile of RDN at 1, 3, and 6 months.

### Pharmacokinetic analysis

The use of animals in the study was approved Ethical committee of Taibah University with approval number EC/TU.123 on 30–08-2025. The rats used in the study weighed between 400 and 600 g and were in good health. There were six albino rats in each of the two groups (control group and test group). The male diseased free albino rats were used for the study. The female and diseased albino rats were excluded from the study. The ICH guidelines were followed for the care and well-being of the animals during the pharmacokinetic study. The animals were housed in a clean room under normal conditions at a temperature of 25 °C with a 12:12-h light-dark cycle, where they had free access to a standard meal and water [14]. Prior the experiment, the animals fasted for 24 h but were offered full access to water. Through a feeding tube, a single dosage of XR5 (equal to 1 mg of drug/kg for the test group) and 150 mg tablets of RDN Actonel® (equivalent to 1 mg/kg for the control group) were given orally. Throughout the sampling process, each animal was carefully identified by tags and kept in wooden crates. Before the dose (t = 0), and then at 0.5, 1, 2, 3, 4, 6, 8, and 24 h after delivery, a 0.25 mL blood sample was extracted from the rat’s tail vein and placed into heparinized micro-centrifuge tubes. Blood was centrifuged at a rate of 5,500 g for 10 min to prepare the plasma samples, which were then aspirated into cryo-vials and kept at 20 °C. RDN was isolated from plasma samples using a liquid-liquid process. 100 μL of acetonitrile was added to a 100 µL aliquot of plasma, vortexed for 20 min, then centrifuged (Hermle Z 220-A) at 4,000 rpm for 25 min. Following centrifugation, an organic layer was removed with the use of a micropipette, and the solvent was dried while being gently sprayed with nitrogen at 45 °C. With 100 µL of the mobile phase, the residue was reconstituted for high-performance liquid chromatography-UV spectrometry analysis.

### Data analysis

Using Microsoft® Office Excel 2010, the concentrations of RDN in plasma samples were determined based on the calibration curve for the range of 200–800 ng/mL. Pharmacokinetic parameters were calculated using Kinetica R version 4.1.1 (Thermo Electron Corporation, United States), a specialized scientific software. Equations 1, 2 were utilized to calculate the highest plasma concentration (C_max_, ng/mL) and the time required to reach the peak plasma concentration (T_max_, h) based on the average collected data. Using Equation 3, the area under the curve to infinity (AUC0- ∞ , ng/mL.h) and the area under the curve to time t (AUC0-t, ng/mL.h) were calculated using the mixed log-linear approach. Equation 4 was employed to compute the Mean Residence Time (MRT, h), Elimination half-life (Kel, h−1), and Clearance (Cl, h−1). AUMC was calculated using Equation 5.

$$c_{\max}=\frac{{FX}_{0}}{V_{D}}\times e^{{-kt}_{\max}}$$ (1)

$$t_{\max}=2.303\log\left(\frac{K_{a}}{K_{e}}\right)\left(K_{a}-K_{e}\right)$$ (2)

$${AUC}_{0-t}=\sum_{1}^{n}\frac{C_{i}+C_{i}+1}{2}\;.\;\Delta t$$ (3)

$$MRT=\frac{AUMC}{AUC}$$ (4)

$${AUMC}_{O-t}=\sum_{1}^{n}\frac{t_{i}\;\left(C_{i}+C_{i}+1\right)}{2}\;.\;\Delta t$$ (5)

where the dose fraction, volume of distribution, and rate constant are given as F, Vd, and k, respectively. The rate constants for elimination and absorption are Ke and Ka, respectively. The drug’s time interval, beginning amount, and ultimate amount are represented by Δt (t2-t1), Ci, and Ci+1, respectively.

## Results and discussions

### Solubility studies

In raft formulations, the calcium is crosslinked with the polymer (XLG) for strengthen the integrity of raft. Major portion of calcium can be utilized in the crosslinking pattern with polymer. The test indicated that the solubility of drug in XR1 to XR9 formulations ranged from 86.5 to 91.7% and 83.9–89.7% in 0.1 N HCl and phosphate buffer pH 5.8 respectively.

### Disintegration and hardness of tablets

The nine formulations had the disintegration times between 28 ± 1.2–51 ± 2.5 s as mentioned in Table 2. The disintegration time pattern of XLG raft-forming tablets composed of XLG and CMC was found to be similar. Formulations with reduced amounts of XLG showed a faster rate of disintegration than those with higher amounts of XLG. The formulation having a higher amount of sodium bicarbonate disintegrates more quickly as compared to the formulation containing a lower amount of sodium bicarbonate. Tablet hardness ranged from 4.18 ± 0.22 to 4.93 ± 0.65 kg/cm^3^. By using pre- and post-compression force analysis, they examined a similar pattern of tablet formulation in the case of hardness [2].

**TABLE 2:** Results of disintegration and hardness of tablets.

Code	Disintegration time (seconds)	Hardness (kg/cm^3^)
XR1	51 ± 2.1	4.23 ± 0.61
XR2	49 ± 2.5	4.54 ± 0.15
XR3	39 ± 1.4	4.29 ± 0.34
XR4	51 ± 2.5	4.78 ± 0.04
XR5	28 ± 1.2	4.93 ± 0.65
XR6	38 ± 1.8	4.19 ± 0.92
XR7	35 ± 2.6	4.34 ± 0.34
XR8	39 ± 2.0	4.18 ± 0.22
XR9	33 ± 3.1	4.55 ± 0.16

### Chemical properties of a raft

The percentage of XLG in the raft determines the integrity and strength of the raft. In the polymeric gel, antacids are additionally trapped to lengthen the neutralization time. The antacids in the formulation instantly neutralize the stomach’s acid and decrease the burning sensation. When tablets of formulations XR1 to XR5 were added, a raft was efficiently developed on the surface of SGF, but when the formulations XR6 to XR9 were added there was not any formation of a raft. The failure of raft formation was due to the lower concentration of XLG polymer and crosslinker in the formulation. The concentration of polymer in the formulation is important factor for the formation of raft. 83% XLG is present in the XR1 formulation, 76% XLG is present in the XR2 formulation, 60% XLG is present in the XR3 formulation and 59% xyloglucan is present in XR4 formulation and 91% xyloglucan is present in XR5 formulation. Due to a better raft crosslinking pattern produced by calcium ions, the XR5 formulation was observed to have a higher concentration of XLG. The XLG raft strength and integrity are improved by the greater concentration of XLG in the raft, which also successfully avoids gastric acid reflux from the stomach into the esophagus. The crosslinking pattern of the raft is facilitated by the calcium ions. Based on their antacid content, the raft-forming formulations have an initial acid ANC, once the formulation is ingested some of the quantity of ANC is used to neutralize the acid pocket [15]. A significant remaining amount got trapped in the raft, as was seen when the rafts were being formed. The ANC of the raft forming tablet was ranged from 3.5 to 8.1. The ANC of the raft was evaluated to determine the raft’s ability to deliver a pool of antacids and maintain the antacid. The highest ANC value of (7.2) was shown by the raft of XR5 formulation, which differed significantly (P-value less than 0.05) from the other formulations. The XR1 formulation had an ANC value of 6.6 (P-value less than 0.05), which was lower than the XR5 formulation but higher than the XR2, XR3, and XR4 formulations, having values 3.1, 2.9, and 2.5, respectively. The failure of the XR1 formulation is due to the presence of excipients in the formulation, this was also reported by Hanif et al in 2020 [2]. The XR5 formulation had a higher ANC than the other formulations due to the higher concentration of antacids. According to earlier studies, antacids are trapped inside the raft, although this hasn’t been measured. A method for measuring the ANC of the raft has been developed. The acid will initially be temporarily neutralized by antacids inside the raft. The ANC and neutralization time of an effective formulation for raft formation must be high. The capacity of the raft to neutralize the acid passing through it was verified by using the neutralization profile. The neutralization duration of each formulation was measured, and the results revealed that the XR5 formulation had the longest neutralization time, i.e. 44.5 min. The XR2, XR3, and XR4 formulations had neutralization times of 33, 22.5, and 37.4 min, respectively. The XR1 formulation could not neutralize the acid. Dettmar et al., published a research article in 2017 that discussed the impact of raft structure on the neutralization profile of formulations that form alginate rafts. Compared to the other formulations, the XR5 formulation containing 83% XLG displayed a higher ANC value and a 44.5-min neutralization profile. This higher ANC value and neutralization profile was seen due to the higher concentration of the effervescent mixture in the XR5 formulation. The XR4 formulation with less XLG percentage displayed a lower ANC value and moderate neutralizing profile [16].

### Swelling of xyloglucan raft

The XLG raft was assessed using a gravimetric technique in simulated gastric fluid (SGF) at pH 1.2 for 60 min to analyze its swelling behavior. As compared to other formulations the XR5 formulation raft showed the best swelling behavior (95.4 ± 2.82% in 60 min) in SGF. This was due to the higher concentration and gel-forming nature of XLG. XLG has a hydroxyl group (-OH) which increases swelling and wettability of the raft. The amount of XLG included in the formulation determines the expansion of raft. In comparison to formulations with lower concentrations of XLG, the formulation with a higher concentration of XLG had a more pronounced swelling pattern. Among the formulations, the raft of the XR3 formulation showed the least swelling (59.2 ± 1.76% after 60 min). Similar swelling percentage patterns of polymeric dosage formulations were also observed by Huanbutta et al. A raft with greater swelling potential tends to remain in the stomach for a longer duration.

### Floating lag time (FLT) and total floating time (TFT)

XR5 showed the highest FLT and the lowest by XR4. Ranges of FLT and TFT of all formulations were 44.4 ± 2.54–56.8 ± 2.86 s and 4.1 ± 0.62–6.2 ± 0.80 h respectively. XLG raft-forming formulations showed a similar FLT and TFT pattern, according to Abbas et al.

### Permeation of RDN

Increased RDN penetration from the drug-loaded raft forming formulation was found during the research. The results showed that the Actonel® (tablet containing RDN) had 35.7% penetration and the raft forming formulation had roughly 67.8% penetration [8]. After oral administration, the synthesized raft forming formulation showed enhanced penetration because of the penetration enhancer.

### Characterization

**FIGURE 1:**
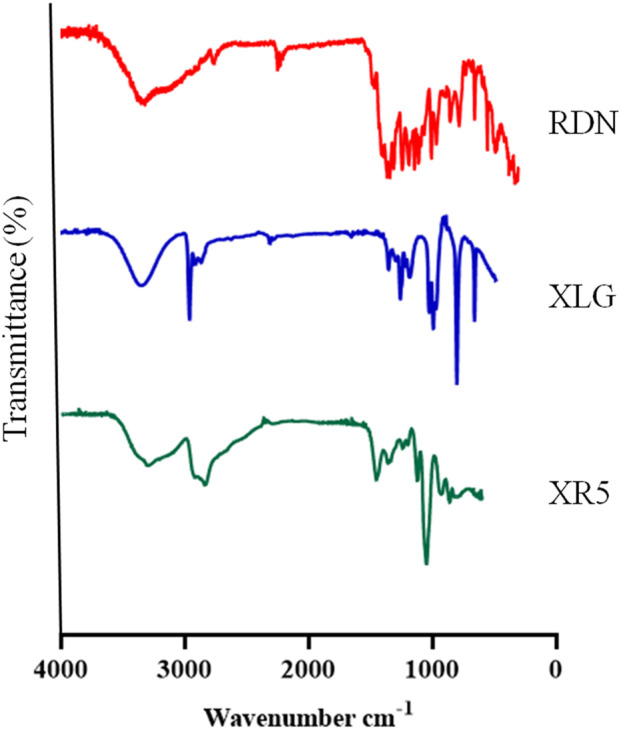
FTIR spectra of RDN, XLG and raft forming XR5 formulation.

**FIGURE 2:**
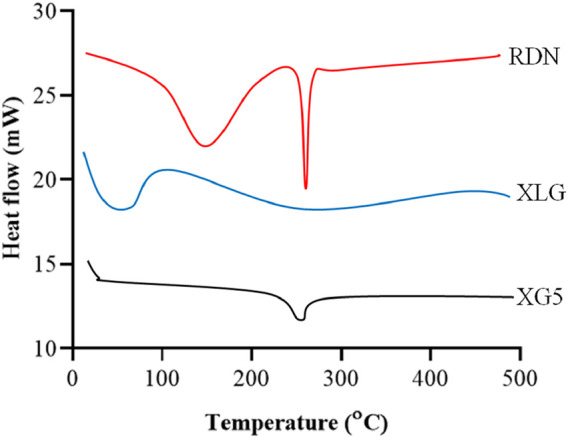
DSC thermograms of RDN, XLG and raft forming XR5 tablets.

**FIGURE 3:**
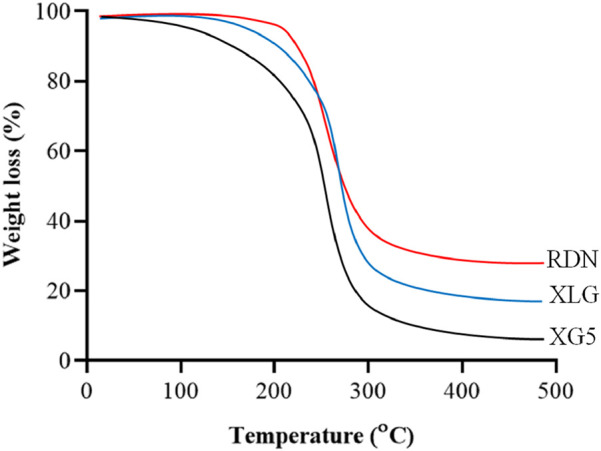
TGA curves of RDN, XLG and raft forming XR5 tablets.

Figure 1 illustrates the FTIR spectra of RDN, XLG, and XR5 XLG raft forming formulation. N-H group stretching, -CH, and C-C group stretching, caused peaks of RDN at 3,395 cm^−1^, 1,604 cm^−1^, and 1,182 cm^−1^ respectively. Due to the stretching in the carbonyl group (C=O) of ester in XLG showed a peak at 1775 cm-1 [17]. There was no chemical interaction between the RDN and the XLG polymer, as evidenced by the raft of the XR5 formulation having peaked at 3,391 cm^−1^, 1775 cm^−1^, 1,604 cm^−1^, 1,182 cm^−1^. These peaks correspond to those of RDN and XLG, respectively. Due to the vibrations of the C-O and O-H groups, the XLG displayed two distinct peaks at 1,176 cm^−1^ and 3,297 cm^−1^, respectively. Figure 2 illustrates the DSC thermogram of RDN, XLG, and XR5 formulation. RDN thermogram exhibited endothermic peaks at 159 °C and 261 °C caused by the loss of crystalline water and which indicated the melting point of RDN [18]. Endothermic peak was observed at 55 °C of the XLG. A single exothermic peak in the XR5 formulation at 261 °C, which showed the loss of crystalline water, and the absence of a peak at 159 °C, which confirmed the uniform distribution and stability of the RDN in the formulation, respectively. Figure 3 illustrates the TGA curves of RDN, XLG, and XR5 formulation. Only a 5% weight loss of XR5 was seen, according to the TGA curves, observed between 40 and 250 °C [19].

### Risedronate release from raft

**FIGURE 4:**
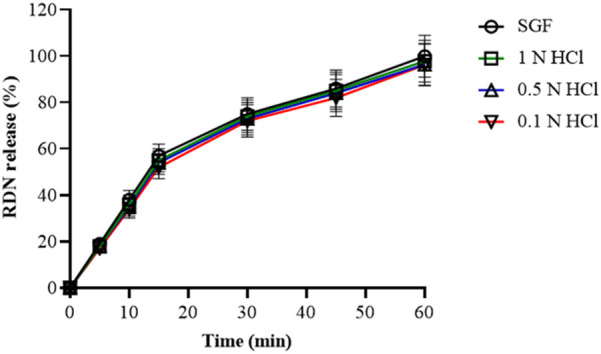
Release profile of RDN from optimized XR5 formulation in different dissolution media (percentage release ± SD, n = 6).

The release of RDN from the modified raft-forming XR5 formulation was tested using the four different acidic mediums to determine the impact of acid strength on raft formation. Dissolution of XR5 formulation was performed in 0.1 N HCl, 0.5 N HCl, 1 N HCl, and SGF for 60 min. RDN was released by the XR5 at percentages of 99.89%, 97.81%, 96.34%, and 96.16% in SGF, 1 N HCl, 0.5 N HCl, and, 0.1 N HCl, respectively, shown in Figure 4. RDN released at a comparable rate across all media, releasing almost 95% of its content in under 20 min. When the XLG raft developed on the top layer when in contact with 0.5 N HCl and 1.0 N HCl solution, the result shows that RDN was mostly present in the aqueous solution. When the RDN came into contact with the 0.5 N and 1.0 N HCl solutions, the RDN was caught in the XLG raft. The RDN then seemed to diffuse out quickly via the foam structures of the XLG raft [2].

### Formulation (XR5) showed non-cytotoxic behavior with Caco-2 cells

**FIGURE 5:**
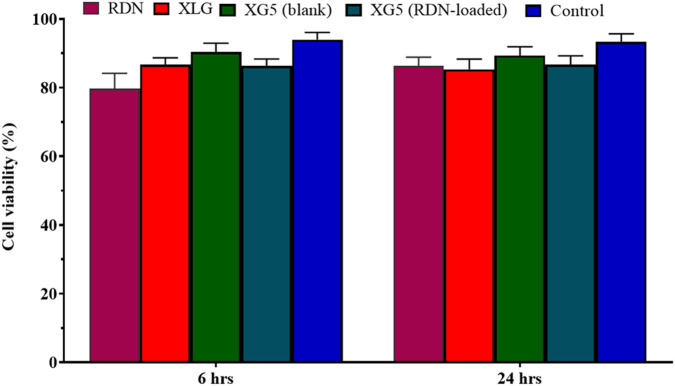
Cell viability assay of RDN, XLG and XR5 formulation.

As Caco-2 cells were treated with or without drug (RDN), as well as with drug dispersion, the XR5 demonstrated up to 88% cell viability when compared to the control (medium only). Figure 5 shows that cells treated with XR5-without dispersion had a viability of 90% after 6 h and 88% after 24 h (P = 0.023). It appears that the toxicity profile of the XR5 formulation for raft based on XLG polymer is extremely near to control, indicating a safe formulation. Our research is consistent with that of Severino et al., who used the Caco-2 and HEPG-2 cell lines to observe the non-cytotoxic behavior of formulations [20].

### Stability studies

The hardness and disintegration time of the XR5 formulation at 0 months were 4.93 ± 0.65 kg/cm^2^ and 28 ± 1.2 s, respectively. Table 3 shows that the FLT and TFT were 54 ± 4 s and 5.5 ± 1.1 h, respectively. RDN percentage release and assay indicated 99.97 ± 4.23% and 97.14 ± 2.31%, respectively. The hardness and disintegration time of tablets after 1 month were 4.81 ± 0.39 kg/cm^2^ and 29 ± 2.2 s, respectively. The raft’s FLT and TFT were 50 ± 5 s and 5.2 ± 1.4 h, respectively. RDN percentage release and assay indicated 95.32 ± 2.89% and 95.28 ± 2.03%, respectively. Tablets’ hardness and disintegration time in the third month were 4.59 ± 1.03 kg/cm2 and 28 ± 2.4, respectively. According to Table 3, the raft’s FLT and TFT were 47 ± 2 s and 4.8 ± 1.2 h, respectively. RDN percentage release and assay indicated 93.46 ± 3.21% and 90.19 ± 1.98%, respectively. Tablet hardness and disintegration time in the 6th month were 4.36 ± 1.09 kg/cm^3^ and 26 ± 1.8 s, respectively. The raft’s FLT and TFT were 46 ± 3 s and 4.5 ± 1.5 h, respectively. RDN percentage release and assay indicated 90.57 ± 0.98% and 88.36 ± 0.57%, respectively [17].

**TABLE 3:** Results of stability studies of XR5 formulation.

Duration in months	Hardness (kg/cm^3^)	Disintegration time (seconds)	FLT (seconds)	TFT (hours)	Release of RDN (%)	Assay (%)
0	4.93 ± 0.65	28 ± 1.2	54 ± 4	5.5 ± 1.1	99.97 ± 4.23	97.14 ± 2.31
1	4.81 ± 0.39	29 ± 2.2	50 ± 5	5.2 ± 1.4	95.32 ± 2.89	95.28 ± 2.03
3	4.59 ± 1.03	28 ± 2.4	47 ± 2	4.8 ± 1.2	93.46 ± 3.21	90.19 ± 1.98
6	4.36 ± 1.07	26 ± 1.8	46 ± 3	4.5 ± 1.5	90.57 ± 0.98	88.36 ± 0.57

### Pharmacokinetic analysis

**FIGURE 6:**
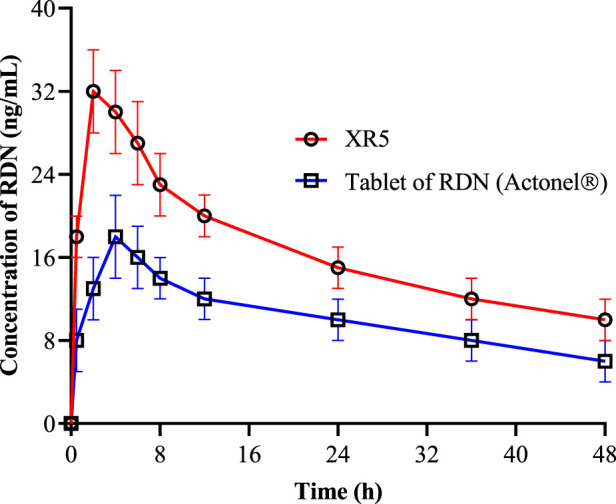
Pharmacokinetics of RDN from tablet of RDN Actonel® (reference) and XR5 raft forming tablets (test) formulation (RDN concentration±SD, n = 6).

The mean plasma concentration-time profiles of RDN following oral administration of suspension of RDN (reference formulation) and XR5 tablets (test formulation) in albino rats were evaluated to study the *in vivo* behavior of the raft-forming RDN tablets (Figure 6). The non-compartmental approach was used to calculate various pharmacokinetic parameters, including Cmax (ng/mL), Tmax (h), AUC0-t (ng/mL.h), AUC0- ∞ (ng/mL.h), AUMC (ng/mL.h), t_1/2_ (h−1), MRT (h), and Kel (h−1). These results are shown in Table 4. Student's t-test was used to statistically analyze the difference between the pharmacokinetic parameters at a 5% level of significance (Table 4). The results showed that the t_max_ for the test formulation was 1.98 ± 0.22 h (P <0.0001) while the t_max_ for the reference formulation was 3.86 ± 0.98 h (P = 0.0003). The XR5 test and reference formulations had peak plasma concentrations of 32.4 ± 2.78 ng/mL and 18.2 ± 2.42 ng/mL, respectively. The test and reference formulations had t_1/2_ of 9.29 ± 2.02 h and 5.29 ± 1.06 h, respectively. The bioavailability of the XR5 was greater than that of the reference formulation, as indicated by the observed AUC_(0-t)_ of the XR5 test formulation, which was 2,987.29 ± 32.12 ng/mL.h and was higher than the AUC_(0-t)_ of the reference formulation, 1,423.29 ± 18.92 ng/mL.h. The test formulation and the reference formulation had AUC (0- ∞ ) values of 5,673.93 ± 38.65 ng/mL.h and 2,476.61 ± 23.61 ng/mL.h, respectively. A rise in AUC, a key metric for estimating bioavailability, may be associated with an increase in the drug’s bioavailability. The highly porous and absorbent nature of the raft was demonstrated by the use of XLG in the formulation. Table 4 shows that the MRT for the test and reference formulations was 17.59 ± 2.98 h and 8.95 ± 1.89 h, respectively. XLG promotes RDN release from XR5, whereas PEG 400 improves RDN penetration into the stomach. When compared to the reference formulation, the test formulation (XR5) had higher bioavailability [2].

**TABLE 4:** Pharmacokinetics Parameters of RDN from XR5 and suspension of RDN (n = 6).

Parameters	Test formulation (XR5)	Reference formulation Actonel® (Tablet of RDN)	P-value
t_max_ (h)	1.98 ± 0.22	3.86 ± 0.98	<0.0001
C _max_ (ng/mL)	32.4 ± 2.78	18.2 ± 2.42	0.0003
t_1/2_ (h)	9.29 ± 2.02	5.29 ± 1.06	<0.0001
AUC_(0-t)_ (ng/mL.h)	2,987.29 ± 32.12	1,423.29 ± 18.92	<0.0001
AUC _(0-_ ∞ ) (ng/mL.h)	5,673.93 ± 38.65	2,476.61 ± 23.61	0.0002
AUMC (ng/mL.h)	77,453.12 ± 58.76	34,567.56 ± 51.78	<0.0001
MRT (h)	17.59 ± 2.98	8.95 ± 1.89	0.0003

## Conclusion

The XLG raft forming tablets was prepared successfully formed and demonstrated effective and porous raft formation. More than 90% of the freshly prepared tablet’s RDN was released within 20 min as it quickly dispersed in the SGF. This dosage form can neutralizes stomach acidity and can keeps gastric pH above 3.5, preventing RDN reflux into the esophagus. The newly developed XR5 showed higher bioavailability than that of the suspension of the RDN. The ideal alternative for delivering RDN orally may be this new XLG raft-forming formulation.

## Data Availability

The raw data supporting the conclusions of this article will be made available by the authors, without undue reservation.
